
JOURNAL INFORMATION
==============================
NLM Title Abbreviation: ScientificWorldJournal
Journal ID: ScientificWorldJournal
NLM Journal ID: 101131163
Journal ID: TSWJ

The Scientific World Journal

ISSN: 2356-6140
EISSN: 1537-744X
Publisher: Wiley

ARTICLE INFORMATION
==============================
PMCID: PMC6084548
PMID: 30147519
DOI: 10.1100/tsw.2001.169
Article ID: 487468
Article version: 1
Subjects: Short Report

Microscopic Photosensitization with Chloromethyl-X-Rosamine on a Subpopulation of Mitochondria Induces Apoptosis in Human Osteosarcoma Cells

Lum Maggie *
Minamikawa Tetsuhiro
Nagley Phillip
Department of Biochemistry and Molecular Biology, PO Box 13D, Monash University, Victoria 3800, Australia
*Maggie Lum: maggie.lum@med.monash.edu.au
Publication date: 2001
Electronic publication date: 2001 Dec 12
Volume: 1
First page: 55
Last page: 55
Copyright: Copyright © 2001 Maggie Lum et al.
Copyright year: 2001
License: This is an open access article distributed under the Creative Commons Attribution License, which permits unrestricted use, distribution, and reproduction in any medium, provided the original work is properly cited.
License URL: https://creativecommons.org/licenses/by/3.0/

==============================
Miami *Nature Biotechnology* Short Reports
*TheScientificWorld* (2001) 1(S3), 55SR
ISSN 1532-2246; DOI 10.1100/tsw.2001.169

### MICROSCOPIC PHOTOSENSITIZATION WITH CHLOROMETHYL-X-ROSAMINE ON A SUBPOPULATION OF MITOCHONDRIA INDUCES APOPTOSIS IN HUMAN OSTEOSARCOMA CELLS

Maggie Lum*, Tetsuhiro Minamikawa, and Phillip Nagley

Department of Biochemistry and Molecular Biology, PO Box 13D,
Monash University, Victoria 3800, Australia
* Maggie.Lum@med.monash.edu.au

**INTRODUCTION.** Chloromethyl-X-rosamine (CMXRos; MitoTracker Red) is a widely used fluorescent dye in imaging mitochondria. We have found that it possesses a strong photosensitizing action (1) and induces apoptosis. As a mitochondrially targeted photosensitizer, CMXRos may provide versatile new applications in studies of mitochondrial roles in cell death. We describe the use of a cell biological approach via microscopic photosensitization with CMXRos by confocal laser scanning microscopy (CLSM) to induce damage in a subpopulation of mitochondria that leads to cell death. This will form the basis for investigation of intermitochondrial communication *in vivo*.

**METHOD.** Human osteosarcoma 143B $TK^-$ cells were loaded with 200 nM CMXRos. For imaging and photoirradiation, an inverted CLSM (Leica TCS-NT) with a Leica PL APO 63X/NA 1.2 water immersion objective lens and equipped with a temperature-controlled chamber maintained at 37°C was used. Photoirradiation was performed under high intensity scanning conditions: xy-scan mode; speed, 1s/frame; zoom, 4; aperture size, 3.65 airy unit; total of 128 scans; laser power around 200 arbitrary unit on the scale of Leica TCS-NT software. For imaging as such, low intensity scanning was carried out to reduce phototoxicity. Properties of cells and mitochondria were monitored at subsequent time intervals after irradiation. The consequences of CMXRos microscopic photosensitization of a subpopulation of mitochondria in individual cells (partial irradiation) were compared to those of cells with their entire mitochondrial population subjected to photoirradiation (global irradiation). Microscopic photosensitization here involved partial irradiation of about half the mitochondria in individual cells.

**RESULTS.** Phosphatidylserine (PS) exposure (Annexin V binding) occurred in less than one hour in about 80% of cells partially irradiated, comparable to that of global irradiation. No uptake of propidium iodide was observed up to 2 h after partial irradiation, indicating that cells were undergoing early apoptosis, but were not necrotic. However, about 20% of globally irradiated cells showed PI uptake. Cytochrome *c* (cyt *c*) was released in more than 80% of partially irradiated cells up to 2 h after irradiation. Global irradiation showed 60% of cells to release cyt *c* before 1 h, and this proportion unexpectedly declined (about twofold) 2 h after irradiation. This anomaly is discussed below. Some of the cells showed apparent non-release of cyt *c* with weak punctate staining localized to mitochondria. Following partial irradiation of cells pre-loaded with both CMXRos and rhodamine 123 (Rh123: to monitor mitochondrial membrane potential, $\Delta\Psi_m$), Rh123 fluorescence declined in both irradiated and non-irradiated mitochondria.

**DISCUSSION.** The PS exposure and cyt *c* release during the early stages of apoptosis demonstrate that partial microscopic CMXRos photosensitization induces apoptosis. From PI uptake studies, none of the partially irradiated cells were necrotic in contrast to the presence of a minor population of necrotic cells following global irradiation. This suggests that the progress towards cell death in partially irradiated cells is slower than that in globally irradiated cells. The observations of cyt *c* release following CMXRos global photoirradiation are anomalous. We propose that this may be due to degradation or conformational changes of cyt *c* that could avoid immunocytochemical detection, subsequent to its initial release brought about by the severe direct mitochondrial damage (1). The loss of $\Delta\Psi_m$ in the non-irradiated mitochondria and cell death observed following partial irradiation could be attributed to possible communication between the irradiated and non-irradiated mitochondria. Signals could be transmitted from the irradiated to the non-irradiated mitochondria directly through the release of soluble factors or indirectly via a downstream caspase-mediated pathway, currently being tested.

**REFERENCE.**

1. Minamikawa, T., Sriratana, A., Williams, D.A., Bowser, D.N., Hill, J.S., and Nagley, P. (1999) J. Cell Sci. 112, 2410-2430
